# Neuropathy of diabetes following initiation of a low-carbohydrate diet: Case report

**DOI:** 10.1016/j.obpill.2024.100115

**Published:** 2024-06-14

**Authors:** Mark Jamal Sahyouni, Luis Anthony Acevedo, Sofia Cristina Rodriguez, Justin Armond Chiles, Nicholas Joseph Pennings

**Affiliations:** Campbell University School of Osteopathic Medicine, Buies Creek, NC, USA

**Keywords:** Case-report, Continuous glucose monitor, Diabetes, Diabetic neuropathy, Low carbohydrate diet, Obesity

## Abstract

**Introduction:**

This case study portrays an unusual case of treatment-induced neuropathy of diabetes (TIND) in a patient with uncontrolled type 2 diabetes (T2D) who achieved rapid improvement in glucose control primarily with dietary intervention. Initial presentation was 50-year-old white male with a long-standing history of obesity and a family history of T2D with a screening glucose level >500mg/dL by glucometer, HbA1c of 14.9%, and initial weight 213 lbs.

**Methods:**

The initial intervention included a low-carbohydrate diet, metformin, and a continuous glucose monitor (CGM). Semaglutide was added after seven days.

**Results:**

His glycemia was within the target range within three weeks. Four weeks after initiation of therapy, he developed TIND symptoms consisting of burning, tightness, and numbness of bilateral feet along with 10/10 pain. At three months, his HbA1c dropped to 6.9% and his weight to 195 lbs. Treatment of his TIND reduced his pain from 10/10 to 2/10.

**Conclusion:**

Whereas TIND is commonly associated with the use of insulin or sulfonylureas, this study adds evidence to the paucity of literature regarding TIND precipitated by dietary intervention.

## Graphical abstract

**Figure:**
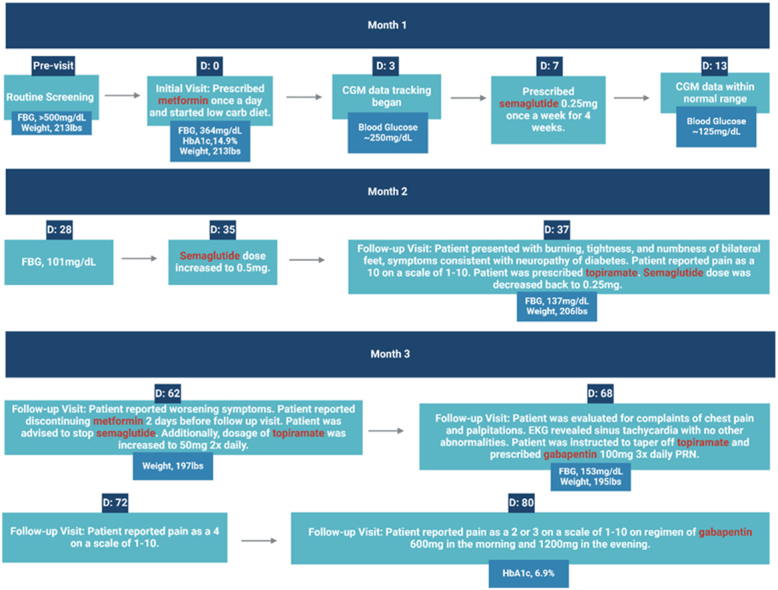

## **1** Introduction

### 1.1 Low carbohydrate diet effects on type 2 diabetes

Type 2 diabetes (T2D) is a chronic disease, impacting over 38 million Americans as of 2021 [1]. The American Diabetes Association (ADA) guidelines identify lifestyle changes as essential for achieving diabetes treatment goals. Standards of care recommendations from the ADA suggest that reduction in overall carbohydrate intake for individuals with diabetes has demonstrated the most evidence for improving glycemia and may be applied to a variety of eating patterns that meet individual needs and preferences [2]. The three dietary interventions recommended by the CDC for patients with diabetes include; counting carbohydrates, the plate method, and limiting portion sizes [3].

Obesity is a common, chronic disease that substantially increases the risk of T2D [4]. While addressing acute metabolic abnormalities should be a first concern in patients presenting with uncontrolled T2D, prioritizing obesity treatment can also provide substantial benefits. Weight loss of just 3–7% improves cardiovascular risk factors and glycemic control while more substantial weight reduction, exceeding 10%, can lead to diabetes remission [5].

Incorporating a low-carbohydrate diet has shown benefit in the management of T2D [6,7]. A very low-carbohydrate diet is commonly defined as 20–50g daily [8]. A study randomly assigned 26 participants with T2D and body mass index (BMI) ≥ 25 kg/m^2^ to either a very low-carbohydrate ketogenic diet (20–50g daily) or a plate method (control group) eating pattern for 32 weeks [6]. Compared to the control group, participants in the intervention group reduced their HbA1c levels an average −0.8% with half of the participants lowering their HbA1c under 6.5% compared to none in the plate method group (P = 0.02) [6]. A comparison of the effects of a low-carbohydrate diet in individuals with obesity (BMI> 30 kg/m2) and normal glucose (91.8 ± 0.72mg/dL) found a 51% reduction in glucose levels over the 56-week study (P < 0.0001) [7].

A short-term, four-day pilot study using a continuous glucose monitor (CGM) compared glycemic control in three groups with T2D; a low-carbohydrate diet group (10% of total energy from carbohydrates), a low-carbohydrate diet followed by a postprandial exercise group, and a low-fat diet group (55% of total energy from carbohydrates) [9]. Researchers found significantly greater glucose control in the low-carbohydrate group when compared to those who were on a low-fat diet (P < 0.001) [9].

### 1.2 CGM use and improved dietary and physical activity compliance

CGMs provide early detection of hyper/hypoglycemia which benefits quality of life and diabetes management [10,11]. Time in range (TIR) and ambulatory glucose profile (AGP) are useful measures of glucose control and correlates with HbA1c [12,13]. Improved TIR is associated with a reduction in diabetic peripheral neuropathy [14].

The use of CGMs can provide individuals with more information about their blood glucose levels in relation to their diet and physical activity. In an 18-item survey of CGM users, 90% felt that CGM use contributed to a healthier lifestyle [15]. 47% percent of CGM users were more likely to go for a walk or do physical activity if they saw a rise in their blood glucose levels, lending to improved blood glucose management. After using a CGM device, 87% percent of participants altered their dietary consumption to best maintain in-target range and 90% of individuals felt their overall lifestyle improved [15]. A randomized control trial examining diabetes outcomes in patients using CGMs found CGM use significantly lower HbA1c, BMI, and improved physical activity adherence after eight weeks compared to patients with traditional glycemic control methods [16].

### 1.3 Treatment-induced complications of diabetes

Common microvascular complications of diabetes include neuropathy, retinopathy, and nephropathy [17]. Treatment-induced complications of diabetes are less commonly recognized causes of microvascular complications. The term “treatment-induced neuropathy of diabetes” (TIND) has been used in the literature to describe the burning, lancinating pain associated with the initiation of insulin therapy in patients with diabetes [18]. TIND has been reported in cases where there are rapid improvements in glycemia not related to insulin use including a case attributed to dietary modification [19]. Since this phenomenon is not well reported in the literature, a formal definition is currently being established. One working definition of TIND is as follows: “1) to have the onset of either neuropathic pain or autonomic dysfunction within eight weeks of a decrease in average glucose values, 2) having neuropathic pain of at least three points on a 10-point Likert scale or autonomic dysfunction that was severe enough to require medical attention, 3) the change in glucose control resulted in a decrease in HbA1C of ≥ two points over three months” [20].

Other complications related to TIND include the exacerbation of retinopathy and nephropathy. While associated with uncontrolled diabetes, worsening retinopathy and nephropathy have been seen in some cases of rapid glycemic change [18,21,22]. The ADA classifies hypoglycemia as a blood glucose <70 mg/dL [23]. Treatment-induced complications of diabetes can occur above this threshold of hypoglycemia, which could be explained by the concept of “relative hypoglycemia”. While still under investigation, relative hypoglycemia is thought to occur when there are decreases in glycemia above the clinical threshold causing hypoglycemic-like symptoms [24]. This may lead to energy-dependent failure of axonal transport, and the release of pro-inflammatory cytokines, and possibly cause treatment-induced complications [20]. There exists a paucity of evidence to provide general information regarding the prevalence of these complications of treatment. A study found that a decrease in HbA1c of 4.4% points over three months increased the risk of developing TIND by over 80% [20].

Risk factors associated with the occurrence of TIND and other complications include a high initial HbA1c, the magnitude of decrease in HbA1c, and the rate of HbA1c reduction [25]. A greater decrease in HbA1c was associated with increased symptoms of TIND. This follows the same pattern seen in the Diabetes Control and Complications Trial where every one percentage point decrease in HbA1c was associated with a 1.6-fold increased risk of worsening retinopathy [25]. Rapid lowering of HbA1c increased the likelihood of microvascular complications, however, the means by which they achieved glycemic control did not impact the occurrence of complications.

Management of TIND usually includes tricyclic antidepressants, gabapentinoids, serotonin-norepinephrine reuptake inhibitors (SNRI), sodium channel blockers, and SNRI/Opioid dual mechanism agents [26,27]. Although self-limiting, the symptoms of treatment-induced complications can take 12–24 months, leading clinicians to pursue symptomatic treatment for their patients [28].

This case is a report of a patient experiencing TIND following rapid improvement of glycemia using CGM and dietary modifications.

## **2** Patient information

The patient is a 50-year-old white male with a history of hyperthyroidism, obstructive sleep apnea, and low testosterone. The patient had obesity for most of his life (highest weight ∼280 lbs). He reports previously losing 80lbs following a low-carbohydrate, ketogenic diet, but slowly regained much of that weight loss in his third decade of life. At the time of his initial visit, the patient's diet includes eating three meals, three-to-four snacks, and consuming 70–80oz of juice/soda daily. Relevant family history includes father with T2D, atrial fibrillation, heart disease and mother with coronary artery disease requiring stents, hypercholesterolemia, and atrial fibrillation.

## **3** Clinical findings

Screening glucose testing via glucometer revealed a blood glucose >500mg/dL on two separate occasions prior to his initial visit. On presentation, the patient complained of fatigue, excessive daytime sleepiness, worsening blurred vision, epigastric abdominal pain, daily headaches, numbness in fingertips, edema, and an isolated episode of palpitations. He had not seen a primary care physician for 10 years before this visit. Blood glucose at the initial visit was 323mg/dL. Initial weight was 214.8lbs with a BMI of 32.9 kg/m^2^. Physical exam findings were otherwise unremarkable.

## 4 Timeline of events

**Fig. 1:**
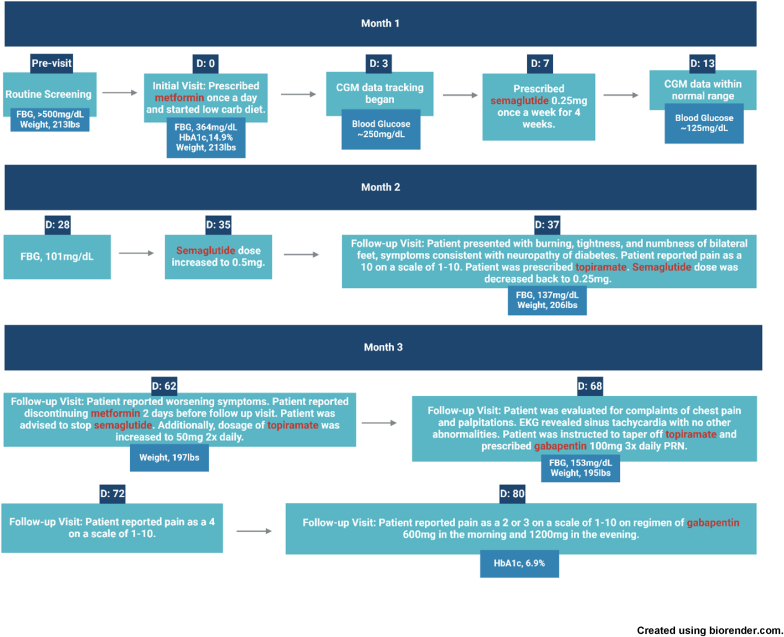
Timeline of events for patient care in TIND case.

## 5 Diagnostic Assessment

Initial lab tests were remarkable for a HbA1c of 14.9% and a C-peptide of 2.6 ng/mL (N = 1.1–4.4 ng/mL). Hepatic and renal function were normal including urine microalbumin. The thyroid panel was normal. Early CGM data revealed a blood glucose level of an average ∼250mg/dL with peaks north of 350mg/dL.

Subsequent diagnostic evaluation after the onset of neuropathic symptoms included a Vitamin B12 level which was 938 pg/mL (reference range: 232–1245 pg/mL) and referral to neurology. EMG showed slight sensory and motor neuropathy with axonal features consistent with diabetic peripheral neuropathy.

## **6** Therapeutic interventions

**Fig. 2:**
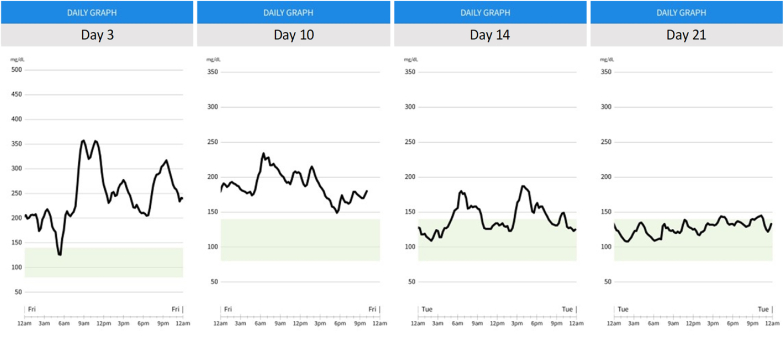
CGM data showing glucose in-target range within 3 weeks.

Through shared decision-making, the patient was started on a low carbohydrate diet (≤20g daily for two weeks and then ≤50g daily afterwards), referred to a dietitian, started on metformin 500mg daily, and provided a CGM with instructions to monitor his post-prandial glucose surges (Fig. 1). After one week, he was prescribed injectable semaglutide 0.25mg weekly. After three weeks, the patient's CGM results were largely within the target range averaging ∼125mg/dL (Fig. 2).

At week four, the dosage of semaglutide was increased to 0.5mg for its weight-loss benefit. Unfortunately, in week five, the patient presented to the clinic with bilateral burning, tightness, and numbness of feet starting a few days prior. The patient reported the pain as a 10 on a scale of 1–10. The patient had lost 8lbs (206 lbs) with a BMI of 31.6 kg/m^2^, and an average fasting blood glucose of 137mg/dL from the prior week via CGM data.

Due to myalgia and neuropathy, the patient was advised to decrease the dose of semaglutide back to 0.25mg once weekly. Additionally, topiramate 25mg PO daily was prescribed to alleviate his neuropathic pain.

At two months, the patient lost an additional 9 lbs (197 lbs) and was at a BMI of 30.2 kg/m^2^. The patient's neuropathy continued to worsen. He complained of feet pain, burning, and a pins-and-needles sensation that substantially disturbed his sleep. Neurological examination was unremarkable including a monofilament exam. The patient was advised to continue dietary changes, discontinue semaglutide, and increase topiramate to 50mg twice daily. The patient elected to discontinue metformin two days prior to this visit because he believed that was causing his pain.

**Fig. 3:**
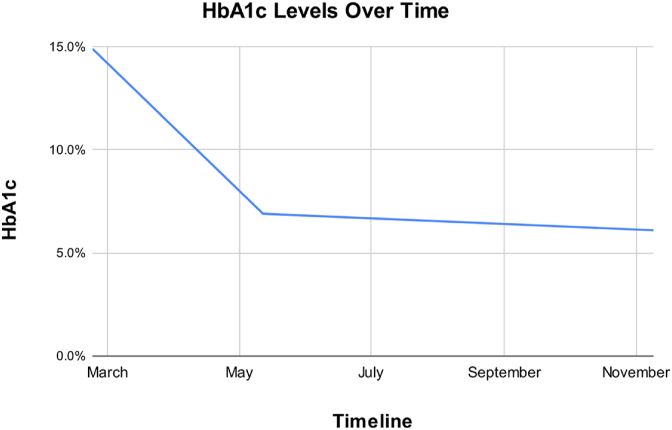
HbA1c levels over 9 months.

One week later, the patient was evaluated for complaints of chest pain and palpitations, along with a reported increase in resting heart rate by 10 bpm. Electrocardiogram revealed sinus tachycardia with no other abnormalities. He continued experiencing a tingling sensation in his feet that improved after an increased dose of topiramate. At this visit, the patient was instructed to taper down from topiramate and was prescribed gabapentin 100mg 3x daily as needed for his neuropathy which subsequently reduced his neuropathic pain to 4/10. Adequate pain control was achieved with titration of the gabapentin dose to 600mg in the morning and 1200mg in the evening. At ten weeks, fasting glucose was 152, HbA1c was 6.9% (Table 1, Fig. 3).

**Table 1:** HbA1c over the course of 9 months.

	Initial	3 Months	9 Months
HbA1c	14.9%	6.9%	6.1%

## **7** Follow-up and outcomes

After one year, glucose remained well controlled off of diabetes medication. He initially lost 27 lbs following a low-carbohydrate diet but regained the weight when he discontinued the restrictions. His fasting glucose was 114mg/dL and his HbA1c was 6.2%. He had no evidence of retinopathy or nephropathy and his neuropathy continued to improve on gabapentin. The patient met the diagnostic criteria for diabetes remission with HbA1c of <6.5% for at least three months after discontinuing all diabetes medication [29].

## 8 Discussion

This report presents a patient with obesity and T2D who experienced TIND following rapid improvement in glycemia primarily utilizing a low-carbohydrate diet, short-term diabetes medication, and CGM monitoring. His initial HbA1c of 14.9% dropped to 6.9% in less than three months. This case exemplifies the potential impact that CGMs and diet can have in the treatment of diabetes. Diabetes self-management and medical nutritional therapy are foundational for achieving treatment goals in diabetes [2].

The absolute amount and the rate of blood glucose-lowering are positively associated with the development of TIND, especially in those with decreases in HbA1c greater than 2% [30]. Diet-induced neuropathy has been rarely recorded in the literature and suggests this is an uncommon presentation for TIND. Our patient's treatment also included starting doses of metformin and semaglutide, drugs that do not cause hyperinsulinemia.

Complications of the aforementioned medications used in the case, including semaglutide, have been reported to cause microvascular complications (i.e., retinopathy). One group investigated this relationship and found a significant difference in the incidence of retinopathy in the treatment group with semaglutide (3%) vs placebo (1.8%). They also found those who experienced retinopathy were more likely to have a previous history of retinopathy, higher HbA1c, longer diabetes history, and were more likely to use insulin [30]. However, glucagon-like peptide-1 receptor agonists have not been directly implicated in causing TIND. Liraglutide showed potential neuroprotective benefits by reducing neuroinflammatory cytokines, specifically interleukin-6 in the early stages of neuropathy [31].

Association between metformin use and TIND has been reported. A cross-sectional study by Pan et al. found a greater prevalence of TIND in patients with T2D taking metformin especially with prolonged use and higher doses [32]. A comparison of patients with T2D treated with metformin versus non-metformin treated patients found a higher incidence of TIND in the metformin group especially with higher doses (>2g), longer duration (>4 years), and low vitamin B12 levels [33]. Yang et al. also found correlation of neuropathy associated with metformin use at high doses (>2g) and for a longer duration [34]. In contrast to those studies, this patient's metformin use was at a low dose, limited to two months, with a normal vitamin B12 level (938 pg/mL, reference range: 232–1245 pg/mL). While the medication doses were low, the potential contribution of medication to the development of TIND in this patient cannot be excluded.

In six months, the patient lost a total of 31.6 lbs, 14.7% of his body weight, and decreased his BMI from 32.9 kg/m^2^ to 28 kg/m^2^. An interesting observation in this case was the contribution of CGM to adherence to diet therapy, improving both glycemic control and weight loss. He was initially prescribed starting doses of metformin and semaglutide, but these were discontinued after two months, while CGM and diet therapy were continued. This enabled the patient to maintain glycemic control and continue to lose weight. Semaglutide is used in the treatment of diabetes and obesity at higher doses than prescribed here. Initial weight loss may have benefited from the weight-reducing effects of semaglutide and metformin, but the remaining weight loss is attributable to CGM and diet therapy.

Obesity plays a formative role in the development of diabetes. Taking a weight-centric approach to the treatment of diabetes improves glycemic control and, with substantial weight loss (>10%), can result in diabetes remission [35]. Low carbohydrate diets can have a profound effect on glycemic control. The introduction of potent diabetes medications that, when combined with lifestyle changes, can produce both dynamic glycemic control and meaningful weight loss, however, clinicians should be mindful of the risk of related microvascular complications. This case exemplifies that risk. While glycemic control remains an essential component of diabetes management, more research is needed to identify those at increased risk of microvascular complications along with identifying effective measures to mitigate that risk.

## 9 Patient Perspective

After discovering his glucose was over 500mg/dL, the patient decided to seek treatment. He was interested in pursuing a low-carbohydrate diet since he was familiar with it, and it had worked for him in the past. The patient described his experience with CGM as extremely helpful because it allowed him to quickly recognize which foods raised his glucose. He emphasized that CGM positively impacted his ability to make dietary decisions because “visualizing it really helped manage it.” When he was first diagnosed with diabetes, he stated he was asymptomatic. When his glucose was under control he began feeling as if someone was “pinching” him all over his body. He did not experience significant relief until he achieved higher doses of gabapentin.

## Informed consent

The patient in this case provided informed consent to be included in this case report.

## Author contribution

MS, SR, LAA, and JC were involved in initial drafts, MS, SR, and NP provided edits and updates, all reviewed and approved final submission.

## Ethics review

Written consent was obtained from the patient to publish this report.

## Source of funding

Beyond payment to the research staff by Campbell University, this research did not receive any specific grant from funding agencies in the public, commercial, or not-for-profit sectors.

## Declaration of Artificial Intelligence

During the preparation of this work the author(s) used did not use AI.
